# Exploring surface water as a transmission medium of avian influenza viruses – systematic infection studies in mallards

**DOI:** 10.1080/22221751.2022.2065937

**Published:** 2022-05-03

**Authors:** Ann Kathrin Ahrens, Hans-Christoph Selinka, Thomas C. Mettenleiter, Martin Beer, Timm C. Harder

**Affiliations:** a Institute of Diagnostic Virology, Friedrich-Loeffler-Institute, Isle of Riems, Germany; b Section II 1.4 Microbiological Risks, German Environment Agency (UBA), Berlin, Germany; c Friedrich-Loeffler-Institute, Isle of Riems, Germany

**Keywords:** Avian influenza, transmission, high pathogenicity, surface water, spread, mallard

## Abstract

Mallards (*Anas platyrhynchos*) are an abundant anseriform migratory wild bird species worldwide and an important reservoir for the maintenance of low pathogenicity (LP) avian influenza viruses (AIV). They have also been implicated in the spread of high pathogenicity (HP) AIV after spill-over events from HPAIV-infected poultry. The spread of HPAIV within wild water bird populations may lead to viral contamination of natural habitats. The role of small shallow water bodies as a transmission medium of AIV among mallards is investigated here in three experimental settings. (i) Delayed onset but rapid progression of infection seeded by two mallards inoculated with either LP or HP AIV to each eight sentinel mallards was observed in groups with access to a small 100 L water pool. In contrast, groups with a bell drinker as the sole source of drinking water showed a rapid onset but lengthened course of infection. (ii) HPAIV infection also set off when virus was dispersed in the water pool; titres as low as 10^2^ TCID_50_ L^−1^ (translating to 0.1 TCID_50_ mL^−1^) proved to be sufficient. (iii) Substantial loads of viral RNA (and infectivity) were also found on the surface of the birds’ breast plumage. “Unloading” of virus infectivity from contaminated plumage into water bodies may be an efficient mechanism of virus spread by infected mallards. However, transposure of HPAIV via the plumage of an uninfected mallard failed. We conclude, surface water in small shallow water bodies may play an important role as a mediator of AIV infection of aquatic wild birds.

## Introduction

Avian influenza A viruses (AIV) are classified in the *Orthomyxoviridae* family. Their octo-segmented single-stranded RNA genome of negative polarity encodes at least 10 structural viral proteins, including the membrane glycoproteins hemagglutinin (HA) and neuraminidase (NA) [1–4]. Based on their antigenicity 16 different HA and 9 NA subtypes can be distinguished [1]. According to their virulence in experimentally infected chickens AIV reveals an either low (LP) or high (HP) pathogenicity phenotype [1,5]. Until today, naturally occurring HP phenotypes are restricted to the subtypes H5 and H7 [5].

In 1996, a flock of domestic geese in the Chinese Guangdong province was identified to be infected with HPAIV termed A/goose/Guangdong/1/1996 (H5N1) [6,7]. Descendants of this virus later became known as the Goose/Guangdong (gs/GD)-lineage [8]. While wild aquatic birds are a long-established natural reservoir for all different LP AIV subtypes [2], they became first infected with gs/GD HPAIV due to spill-back events from poultry in 2003 [9]. Domestic ducks fattened in harvested rice paddies in Southeast Asia probably played a prominent “seeding” role [9,10]. Gs/GD HPAIV remained endemic in poultry populations in several countries in the region and elsewhere [10–13]; continued circulation with frequent host species switches stimulated viral diversification into several phylogenetically distinguishable clades and numerous genotypes. Transmission events at wild bird-poultry-interfaces have continued [14–17]. HPAIV-infected migrating waterbird populations, in addition to (prohibited) transboundary trading, are an important vector of long-distance transmission resulting in several “out-of-Asia” waves of viral escape from endemic regions in the last 15 years hitting Europe [10,18–20] North America [21], and Africa [10,15,22] with unprecedented impact.

Before 2002, only very few sporadic HPAIV infections of restricted geographic expansion were known to have occurred in wild birds [14,23]. Almost all of these cases were traced back to spill-over infections from likewise rarely occurring HPAI outbreaks in poultry, all unrelated to the gs/GD lineage. The emergence of the gs/GD lineage of HPAI viruses has caused a paradigm shift: promoted by the grossly expanded poultry production in Southeast Asia and elsewhere and fostered by the ability of many of the gs/GD lineage viruses to infect Pekin ducks, and other dabbling duck species, asymptomatically or only causing mild clinical signs, these viruses quickly established an endemic status in poultry. For the first time in the history of HPAIV migratory wild birds were assigned a pivotal role as vectors in the transboundary and transcontinental spread of HPAIV. Due to the possible widespread presence of HPAIV in wild bird habitats and spill back by direct or indirect contacts from infected wild birds into poultry the vicious cycle of mutual virus transmission at the wild bird bird-poultry interface had been closed.

It has been estimated that a single HPAIV infected duck can shed 10^10^ EID_50_ doses into the environment within a range of 24 h [24,25]. As fecal–oral transmission chains are highly effective this would be theoretically sufficient to infect another 10^6^ ducks assuming, conservatively, the minimal infectious dose per animal would amount to 10^3^–10^4^ EID_50_ [24]. Dispersion of virus-contaminated fecal matter and oropharyngeal excretions in surface waters is expected to potentiate transmission efficacy: (1) Depending on the AI virus strain but also on various physico-chemical properties of the water body such as temperature, pH, salinity, sedimentation rates, presence of biologic compounds and so on, the infectivity of virus particles shed into such water is retained for astonishingly long periods of up to several months [26]. Conversely, higher water temperatures (22°C), alkaline or acid pH and high salinity inactivate viral infectivity within hours to days [26–30,31]. Furthermore, the presence of invertebrate animals (clams, snails, shrimps, insects) may modulate the retention time of infectious AIV in surface water and sediments [26,32–34]. (2) Several studies succeeded to detect AIV infectivity in free-floating natural water bodies as well as in their sediments [35–38]. (3) Dabbling ducks and swans feed on or closely below the surface of shallow water bodies and may come into contact with dispersed viral infectivity. Viruses deposited in sediments of freshwater bodies over winter have been reported to close a gap in the avian influenza infection cycle of aquatic wild birds in North America [39,40].

So far, most reports on the natural biology of AIV in metapopulations of wild birds concentrated on the birds themselves. Few studies have actively examined the putative role of the environment acting as a source for virus spread and persistence [41,42] and even less targeted water as a transmission medium [43,44]. Here we focussed on experimental infections using surface water as a medium of virus transmission among small flocks of mallards. We show that minute amounts of viral infectivity suspended in surface water are sufficient to start infections in mallard groups and that the presence of a pool versus a bell drinker as a water source has a synchronizing effect on the course of both LP and HPAIV infections.

## Materials and methods

### Virus origin and propagation

Avian influenza virus isolates A/mallard/Germany/AR511/2018 (H4N6) and A/barnacle goose/Germany-SH/2020AI02167/2020 (HP H5N8) were selected from the virus repository at Friedrich-Loeffler-Institute, Germany. Both viruses were cultivated in embryonated chicken eggs in a BSL-3 environment as previously described [45]. Infectivity (TCID_50_) was titrated in MDCK-II cells. Cells (suspension of 100 µL) were either seeded a day before or simultaneously (i.e. together with virus) into 96 well plates. Cell counts were adjusted to 10^5^ cells/ml. Growth medium consisted of MEM supplemented with 2% FKS, Penicillin/Streptomycin (100 U/mL, final) and TPCK-Trypsin (2 µg/mL, final). In case pre-seeded cells were used, the growth medium was discarded before adding 100 µL of virus dilution per well. Virus was diluted in log_10_ steps in a growth medium without FCS supplementation. Cytopathic effects were scored by light microscopy, and, in addition, cells were stained by an immune peroxidase assay as previously described [46]. Briefly, infected cell cultures after 3–5 days of incubation at 37°C were heat fixated at 80 °C for four hours. For primary staining, the cells were incubated with 50 µl of a 1:50 dilution PBS with 0.005% Tween 20 (PBST) overnight at 4°C of the nucleocapsid protein-specific monoclonal antibody 890 (H16-L20-5R5, FLI Biobank). After discarding the supernatant and washing the cells three times, the secondary antibody (50 µL of a 1:500 dilution in PBST of goat anti-mouse IgG (H/L):HRP, Bio-Rad Laboratories GmbH, Feldkirchen, Germany, Lot#151517), was added for 1 hour at 37°C. 3-Amino-9-Ethylcarbazol was used as a chromogene. Between incubation steps, plates were washed three times using 150 µL of PBST. The TCID_50_ was calculated using the Kärber formula as cited in [47].

Virus isolation from selected clinical or environmental samples was carried out in embryonated chicken eggs as detailed elsewhere [48].

## Animal experiments

### Ethical statement, animal rearing

All animal experimental work has been licensed by the animal ethics committee of the Federal State of Mecklenburg-Vorpommerania (LALLF 7221.3-1-023/21, TV „FLI 08/21: Aviäre Influenza in Oberflächenwasser). A total of 80 subadult male and female mallards (10–13 weeks of age) were purchased from a commercial breeder in Germany. At FLI, birds were housed on the floor in BSL-3 stables for acclimatization for 2 weeks together in two large groups of 40 birds each. Blood (0.5 mL) was collected from 20 randomly selected animals on arrival. When separating into smaller groups of up to 10 birds in stable units of about 11 m^2^, a balanced gender ratio was observed. No bedding material was used. Instead, elevated foot-protecting rubber slabs were laid out in parts of the unit to provide a dry area; floors were cleaned on a regular daily base using water. Depending on the experiment, drinking water was provided to the animals either only via bell drinkers or with an additional freshwater pool of 80 cm in diameter into which tap water was added to a fill height of 20 cm representing 100 L. Water in bell drinkers was replenished daily, pool water was fully replaced every 4 days but topped up with fresh water daily. Tap water is tested regularly in accordance with legal requirements. Chlorination or other disinfectant treatment of tap water is not regularly practiced in Germany. Pelleted commercial duck feed to which wheat corns were added was provided ad libitum. A 12-h day–night light cycle was provided.

### Statistical analysis

Differences in clinical scores between the groups were assessed by an unpaired *t*-test with Welch's correction employing the statistical analysis tool of the GraphPad Prism software version 7 (GraphPad Software, San Diego, CA, USA).

### Sampling schemes

Swabs: Each animal was sampled on a daily base. Swab samples were obtained orally, cloacally and from the plumage by rubbing the swab repeatedly over the feather-covered breast of the birds. The swabs were stored until further processing – in cell culture growth medium supplemented with penicillin and streptomycin but lacking FCS at −80°C.

Feathers: A secondary flight feather was collected from the animals at days 4 and 9 in the first round of experiments and at days 0, 4, 9, and 13 for part 2.

Sera: Blood samples for serum analysis were collected from 20 randomly chosen animals at arrival at the FLI. Additional blood samples were collected from each bird when the experiments commenced and ended. The sera were heat inactivated at 56°C for 2 h and stored at 4 °C.

Water: Water amount of roughly 15 mL was collected daily either from bell drinkers or pools depending on the group.

Organs: Animals that died spontaneously during the infection or had to be euthanized when defined humane endpoints were reached were necropsied and tissue samples from lung and brain were taken for virological analyses.

### RNA extraction

RNA was extracted from swabs by the Macherey-Nagel NucleoMag® VET-Kit (#744200.4) according to the instructions of the manufacturer by using the Biosprint 96 extraction robot (Qiagen, Hilden, Germany). Elution was achieved in 100 µL of the supplied elution buffer. Cones from feather samples were cut longitudinally and then across into cell culture medium and then shred in a tissue-lyzer for 2 minutes at 300 Hz; supernatant was then extracted as described above. Similarly, up to 20 mg of an organ sample was processed uncut in a cell culture medium in a tissue lyzer. Clarified supernatant was used to extract RNA manually via the QIAamp Viral RNA kit (Qiagen, Hilden, Germany). Water samples were extracted using the Zymo Environ Water RNA Kit (Cat# R2042, Zymo Research Europe GmbH, Freiburg, Germany) including also the viral enrichment step as described in the kit’s manual. After the viral enrichment step, the samples were stored at −80°C until further processing. In some cases, samples were also extracted via the Macherey-Nagel NucleoMag® VET-Kit (#744200.4).

### Real-time RT-PCRs

All samples were analysed by real-time RT–PCR (RT-qPCR). The samples were examined using a generic RT-qPCR targeting a fragment of the NP gene [49] supplemented with internal control (IC-2 [50]) to control for PCR inhibitory reactions. Samples with a Cq-value <40 were considered positive. Samples >40 were considered negative, if the Cq-value of the IC-2 target was 30 (± 2). All RT-qPCRs were carried out using the AgPath-ID™ One-Step RT–PCR Kit (Applied Biosystems™, AM1005) in a CFX96™ Real-Time-System C1000™ Thermal Cycler or the C1000Touch™ Thermal Cycler (BioRad, Munich, Germany). Cycling conditions were as follows: Reverse transcription 45°C, 10 min; Taq pol activation 95°C. 10 min, and 45 cycles of denaturation (95°C, 15 sec), annealing (56°C, 20 s), elongation (72°C, 30 s).

### Serology

All serum samples were tested by ELISA for influenza-A-specific antibodies. Generic, influenza-A-nucleocapsid protein-specific antibodies were detected by the ID Screen® Influenza A Antibody Competition assay (ID-Vet, Grabels, France). Samples testing positive in the generic NP-ELISA were further tested in an influenza-A-H5-specific ELISA (ID Screen® Influenza H5 Antibody Competition, ID-Vet).

## Results

### Virus spread in stabled mallard groups with or without a surface water pool (experimental infection setting 1)

**Figure 1.**
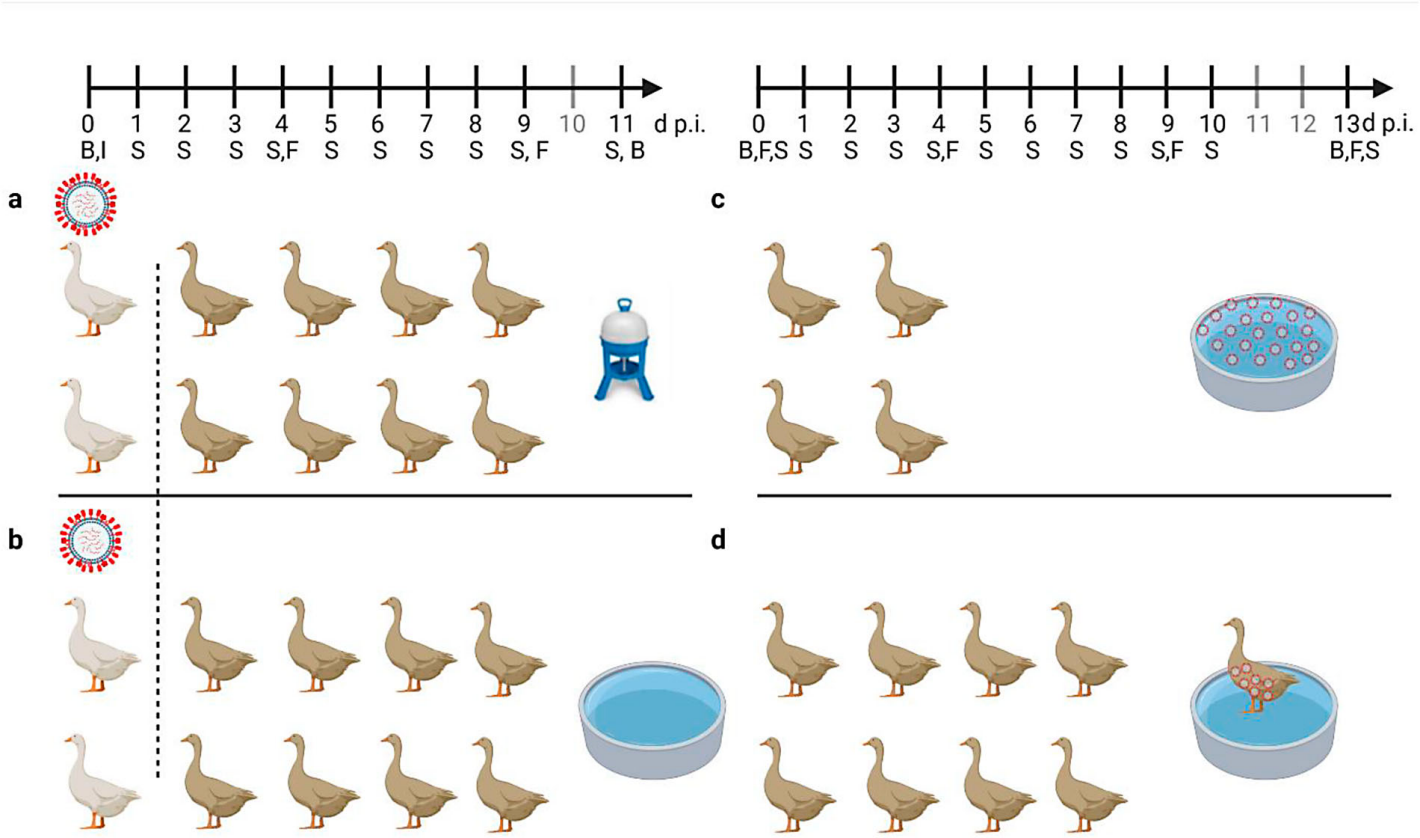
General settings of infection experiments in mallards using two infected “seeder” ducks (light colour), eight contact mallards (dark colour) and two different sources of water (a) bell drinker versus and (b) pool. Seeder ducks of (a) received LP H4N6 while those of (b) were inoculated with HP H5N8. Setting (c) provides artificially contaminated pool water to four ducks as a source of infection and (d) uses a non-infected “seeder” duck with virus-contaminated plumage as contact to eight contact mallards. Created with BioRender.com. An observation time line is indicated at the top; I – Inoculation of two seeder ducks (a, b) B – blood sample, S – Swab sample (oropharyngeal, cloacal, plumage), F – Feather sample (secondary flight feather); red circled dots indicate presence of virus in water (c) or adhering to plumage (d).

Four groups each of 10 mallards were assigned to inoculation experiments using an LPAIV of subtype H4N6 and an HPAIV of subtype H5N8, clade 2.3.4.4b, respectively. In contrast to standard inoculation experiments, only two mallards per group were inoculated oro-oculonasally with 10^6^ TCID_50_ in 1 mL of the respective viruses. These birds were kept separate for 1 day and provided as virus seeder a source of infection for the eight naïve mallards of each group. While groups were housed in identical stable units, one group each of the respective viruses was offered a small swimming pool of 100 L of water adjusted with solid NaCl to a salinity of 0.15% (w/v), i.e. 1500 ppm, which equalizes the salinity of the Baltic Sea at its southern coast line. The other stable unit was equipped with bell drinkers as the sole drinking water source (Figure 1a). Ducks in the pool groups made extensive use of the pool with often up to five birds simultaneously dabbling and diving in the pool. Birds that were offered bell drinkers drank frequently and made surrogate movements to imitate dabbling and washing on surface water. The spread of infection in each group was followed by daily swabbing and by selected serum sampling before infection and at the end of the experiment.

**Figure 2.**
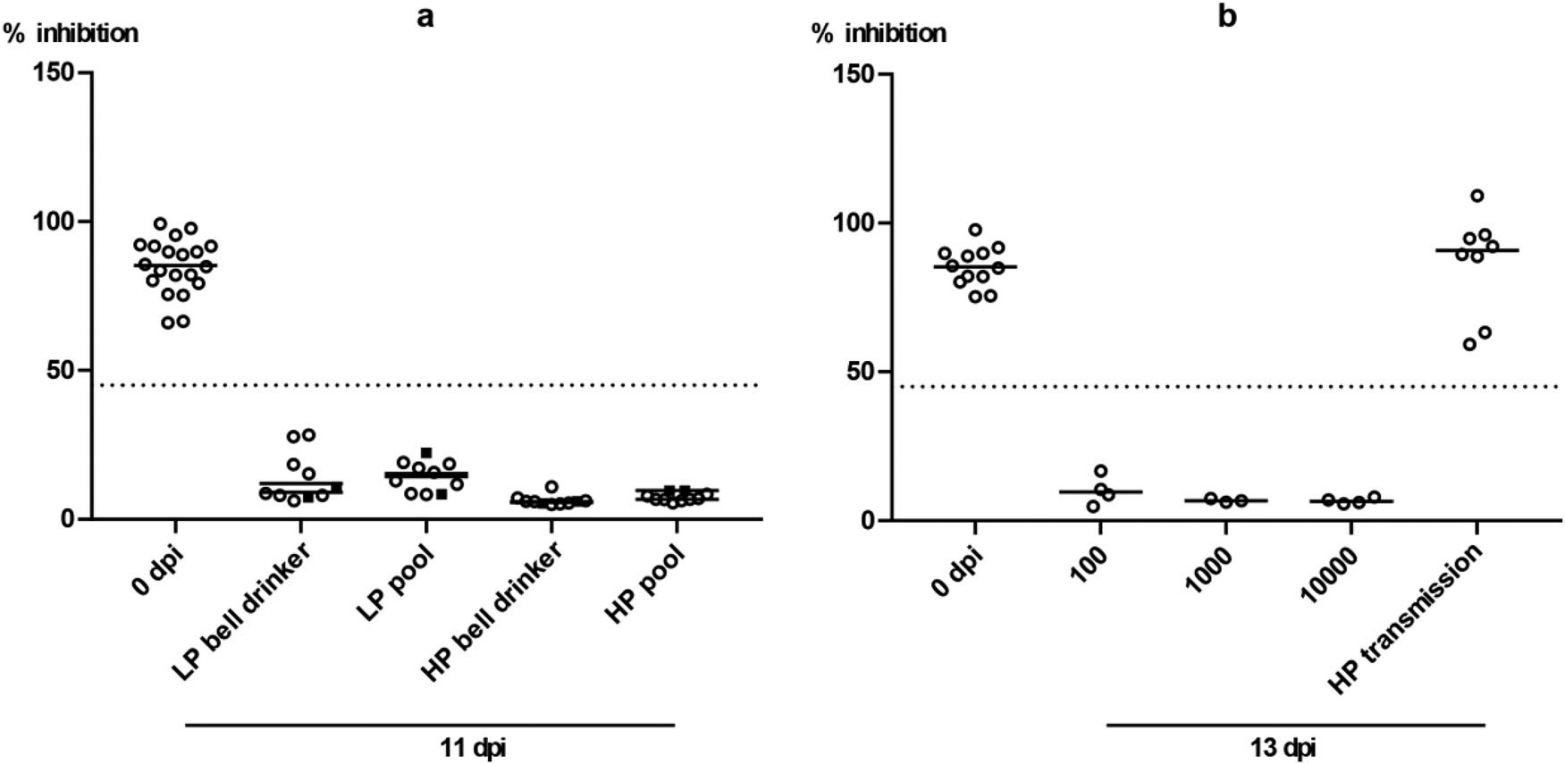
Seroconversion at the beginning and at the end of the observation period (11 or 13 dpi, respectively) against AIV nucleoprotein as measured by blocking ELISA in inoculated (black squares) and naïve (open circles) mallards exposed to LPAIV H4N6 (LP) or HPAIV H5N8 (HP) via inoculated “seeder” ducks (a) or via pool water (b) artificially loaded with HPAIV H5N8 at 100, 1000 or 10,000 TCID_50_ L^−1^. The dotted line represents the ELISA threshold. % inhibition indicates the blocking efficacy of mallard sera.

Clinical scoring during the observation period of 11 days inconsistently revealed minor clinical signs of disease in very few individual mallards in the H4N6 groups (supplemental Figure 1a and b). The same mild progression of the infection was evident in the HPAIV exposed bell drinker group (supplemental Figure 1d). In contrast, in the HPAIV pool group two contact mallards developed a slowly progressing neurological disease consisting of disorientation, head tilting, ataxia and, finally, somnolence. Statistical analysis showed a significant difference between the two HP groups (bell drinker vs pool; *p* = 0,0019). One bird died overnight from 6 to 7 dpi while the other was removed from the experiment at 7 dpi when humane endpoints were reached (supplemental Figure 1c). Postmortal brain tissue samples of each mallard were obtained and harboured high HPAIV H5N8 viral loads ranging at Cq 15–23 (not shown). Four further mallards in this group showed very mild and transient (often only for a single day) clinical signs (supplemental Figure 1c). While an asymptomatic to mild course of the infection has been expected for the LPAI virus groups, the low frequency and mild nature of clinical affection of 18 of 20 HPAIV-exposed mallards were surprising. Seroconversion in all mallards finally surviving until day 11 of the experiments indicated that all naïve mallards had been infected with the respective LP and HP viruses (Figure 2a).

Evidence for productive infection in the inoculated and contact mallards was also obtained from analysing viral RNA loads in swab samples (Figure 3). The four LP- and HP-inoculated mallards started oropharyngeal virus excretion at day 1 post-infection (dpi). Cloacal virus excretion commenced with 1–6 days of delay (Figure 3(1)). Swabs of some but not all of the inoculated mallards stayed virus-positive until the end of the observation period (11 dpi) although with receding viral loads (Figure 3(2)). In the bell drinker group of the H4N6 exposed mallards, infection transmitted immediately to contact ducks and peak excretion of viral RNA was reached around days 3–5 (Figure 3(2 a,b)) for oral swabs; similar patterns evolved in plumage and cloacal swabs, although higher RNA loads peaked one day earlier in cloacal swabs. The presence of a pool apparently delayed transmission of H4N6 infection for almost two to four days but then the infection progressed rapidly and viral RNA loads peaked at days 6 and 7. Similar patterns were also measured for HPAIV exposed mallards although here hardly a delayed transmission of virus to sentinels in the pool group was visible (Figure 3(2)). Viral excretion adjourned earlier (at around day 8) in the pool group. No significant difference regarding oral versus cloacal excretion was observed in these groups. Figure 3.(1) Dynamics of influenza A virus infection of eight naïve mallards exposed to two “seeder” ducks inoculated with LPAIV H4N6 (figure subsets a-f) or HPAIV H5N8 (g-l, blue) and housed in the presence of a small swimming pool or a bell drinker only. Qualitative results of viral RNA excreted orally, cloacally, and adhering to the breast plumage (percentage RT-qPCRs-positive mallards per sampled animal in each group) are shown. Black squares indicate inoculated mallards, open circles represent contact ducks. (2) Influenza A virus RNA loads excreted orally (a and b), cloacally (c and d), and adhering to the breast plumage (e and f), by each ten LPAIV H4N6 infected mallards exposed to pool or bell drinker water. A similar experimental set up was run with ten mallards each infected by HPAIV H5N8 (right panels, blue label).

**Figure:**
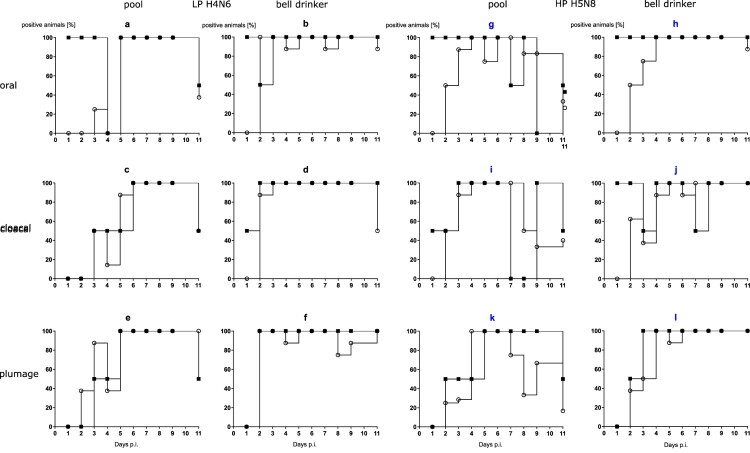

**Figure:**
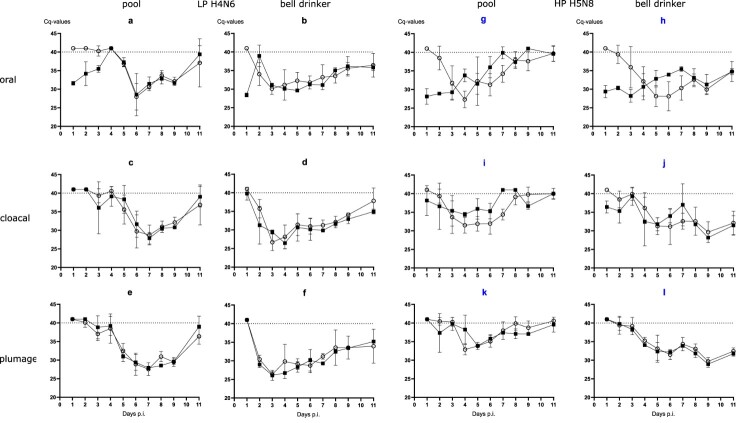

### Approximation of a minimal infectious dose of HPAIV suspended in surface water (experimental infection setting 2)

Concluding from the previous experiment, the water source made available in the stable units might have some influence on the course of an AIV infection in mallards. The transmission of both LP and HPAIV from inoculated seeder ducks to naïve sentinels was possible also in the presence of a small pool resembling a source of brackish surface water. However, direct contact between seeder and sentinel ducks or other indirect contacts (e.g. via contaminated food) could have played a more important role compared to water as a medium of virus transmission. Therefore, we next investigated the contagiousness of brackish surface water artificially seeded with HPAI virus and in the absence of infected seeder ducks (Figure 1c). Three different virus concentrations were used where each litre of a 100-L pool contained 10^2^, 10^3^ or 10^4^ TCID_50_ of HPAIV H5N8. This translates to 0.1, 1 and 10 TCID_50_ per mL. Four ducks were associated with each of the pools and observed for 13 consecutive days.

**Figure 4.**
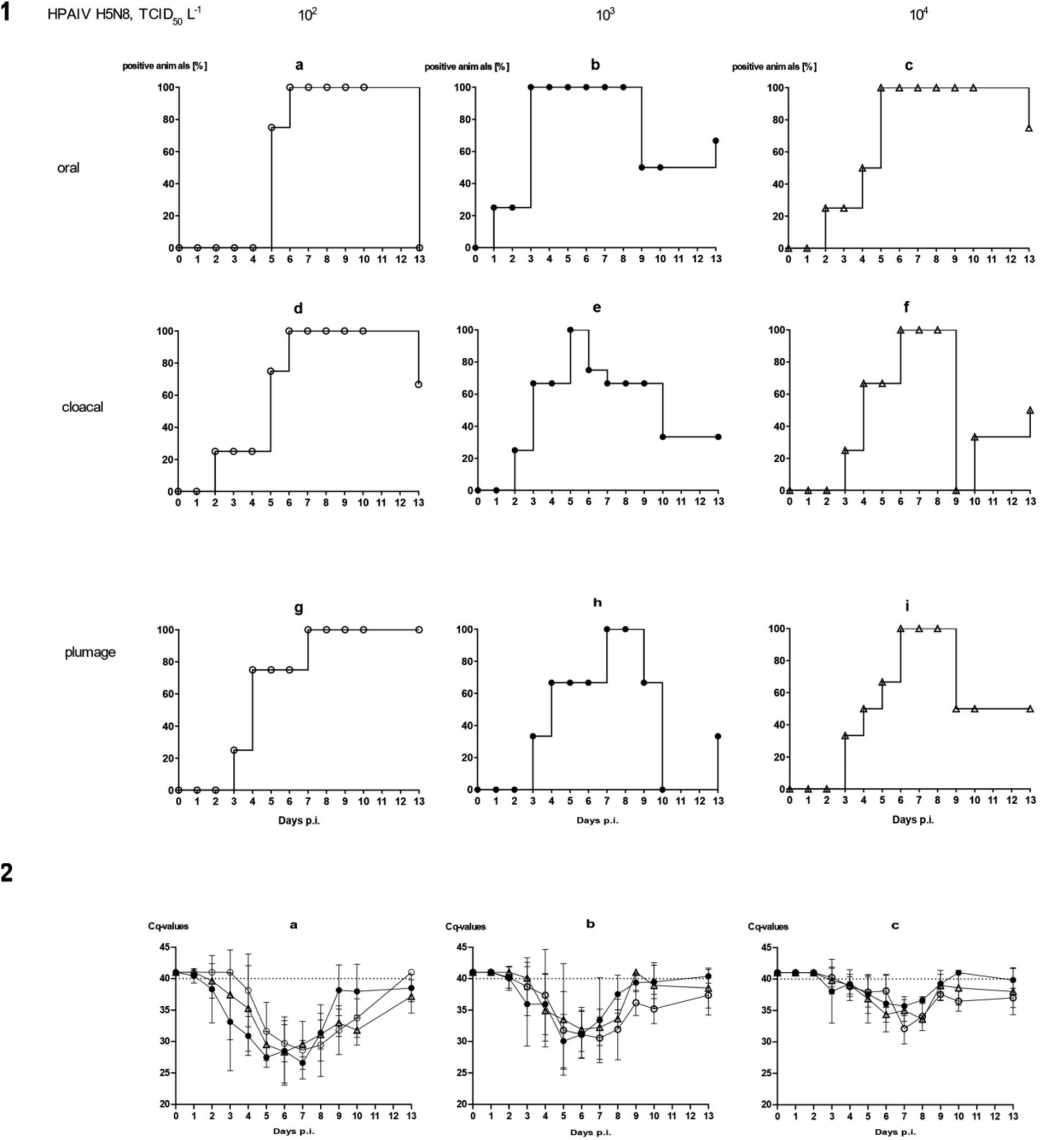
Dynamics of influenza A virus infection of four naïve mallards each exposed to artificially pool water containing 10^2^, 10^3^ or 10^4^ TCID_50_ L^−1^ of HPAIV H5N8. (1) Qualitative results; percentage of mallards excreting viral RNA orally, cloacally, and of RNA adhering to the breast plumage (percentage RT-qPCRs-positive mallards per group) are shown. (2) Quantitative results of viral RNA excretion compared between exposure groups 10^2^ (open circle), 10^3^ (filled circle), and 10^4^ (open triangle) in (a) oral, (b) cloacal and (c) plumage swabs.

With the exception of a single mallard that succumbed to a neurological disease (humanely killed on 10 dpi) in the group exposed to the lowest concentration of HPAIV in the water, no clear clinical signs were observed in other ducks (supplemental Figure 2a–c). Mallard #33 in the 10^3^group had to be removed at 2 dpi after it contracted a severe leg injury (supplemental Figure 2b). Viral RNA loads in swabs showed a delayed onset of infection (Figure 4). In the 10^2^ group it took until 6 dpi until all mallards tested positive (Figure 4(1 a, d)) for oral and cloacal and 7 dpi for plumage swabs (Figure 4(1g)). However, a similar pattern (3–6 days for cloacal and oral swabs [Figure 4(1 e and f)], and 6–7 days for plumage swabs [Figure 4(1h and i)]) of delayed infection was also evident for the groups which were exposed to higher virus titres (Figure 4(1)). Quantitative assessment of virus excretion showed a very similar course of infection independent of the exposure dose in water (Figure 4(2)); from 9 dpi on only marginal RNA loads were detected. Seroconversion against AIV nucleoprotein in all mallards surviving until 13 dpi confirmed RT-qPCR results in that all mallards in all groups became infected.

### A possible role of plumage for transmission of AIV (experimental infection setting 3)

In experimental settings 1 and 2, swabs were also sampled to detect viral RNA adhering to the breast plumage of the mallards. For the collection of material, the swab was rubbed several times carefully down the surface of the breast plumage before being transferred to a transport medium. RT-qPCR revealed identical patterns of viral RNA with slightly lower loads as obtained for oropharyngeal and cloacal swabs (Figures 3(2 e-l) and 4(2 a-c)). The viral RNA detected at the plumage surface could represent contamination from accessing virus-loaded surface water. However, since the plumage was likewise positive for viral RNA in the bell drinker groups, it is more likely to originate from virus-containing oropharyngeal fluids deposited by the individual birds during plumage care and preening. Alternatively, viral RNA on plumage might have been a result of infection of the feather cone epithelia. To test this hypothesis, a small secondary flight feather was plucked in regular intervals (0, 4 and 9 dpi, setting 1; 0, 4, 9 and 13 dpi, setting 2) from a wing, its shaft removed and its conus cut and shredded in a tissue lyzer before RNA extraction. While all feather cones were negative at 0 dpi, the majority of them tested positive at 4 dpi. However, only very low virus loads between Cq 35 and 39 were measured; no differences were seen between LP and HP infected birds (not shown). By 9 and 13 dpi, the majority of feather cones tested negative again. Thus, efficient infection of feather epithelium does not seem to be the source of the viral RNA detected on the plumage.

We selected 10 plumage swabs from a range of high (Cq = 26) to low (Cq = 36) viral RNA loads and subjected them to virus isolation in 11 days old embryonated hen eggs. Isolation was difficult because of the high bacterial burden of the samples. Four swabs yielded infectious virus on days 4–8 (Table 1). This indicates that breast plumage of infected mallards not only harboured viral RNA but, at least in some birds, also carried virus particles that were infection-competent.

**Table 1.** Detection of viral infectivity in selected RT-qPCR-positive plumage swab samples obtained from mallards in experiments 1 and 2.

Animal/experiment	Group	Day (*p*.i.)	Cq value^a^	Virus replication
57/1	LP-Pool	7	29.64	+++^b^
57/1	LP-Pool	8	32.40	++-
74/1	LP bell drinker	3	26.12	- - -
16/1	HP bell drinker	9	29.11	- - -
17/1	HP bell drinker	11	31.24	- - -
23*/1	HP bell drinker	11	31.30	- - -
496/2	HP 10,000	4	35.94	++-
496/2	HP 10,000	6	33.62	++-
382/2	HP 10,000	6	33.91	- - -
363/2	HP 10,000	13	33.48	- - -

*indicates an inoculated animal.

^a^ Cq value of the original sample as measured by generic RT-qPCR targeting the NP gene.

^b^ + indicates virus-positive embryonated chicken egg (three eggs per sample used).

In a third experimental setting, we investigated whether infectious virus can also be carried in the plumage of uninfected ducks. A single mallard (“carrier duck”) was placed for 2 hours in the surface water pool of experimental setting 2 (Figure 1c) spiked with 10.000 TCID_50_ of HPAIV H5N8 per litre. Thereafter the animal was transferred immediately (while “dripping wet”) to another stable unit with a pool of the same size which had not been spiked with virus (Figure 1d). Eight naïve mallard ducks stayed in contact for four hours with this “carrier duck”. Finally, the carrier was removed (after 6 hours of its initial contact with contaminated water) and the remaining eight ducks were observed for 13 days. Within this period none of the contact ducks became infected as judged by consistently negative RT-qPCRs in swabs, lack of clinical signs and failure to seroconvert (Figure 2b) while the carrier duck developed infection and seroconverted as one of the animals used in experimental setting 2. Daily water samples obtained from the pool of experimental setting 3 never tested positive for viral RNA (not shown).

### Presence of moderate to high viral RNA loads in water accessed by infected mallards

Daily samples of water obtained from bell drinkers and pools were tested for NP-specific RNA by RT-qPCR. In contrast to animal swabs, these environmental samples were extracted using the Zymo Environ™ Water RNA Kit. As shown in Table 2 moderate to high RNA loads, represented by low Cq values, were observed in both water sources. In experimental setting 1, particularly high loads were detected in the bell drinker samples; the comparatively small surface of water accessible to ducks via the bell drinker may have produced a concentrating effect (Table 2a). In experimental setting 2, the period and amplitude of viral RNA presence correlated with the initial loading dose of the water (Table 2b). However, RNA was detectable immediately after loading of the pool water only in the group with the highest dose (10^4^ TCID_50_ L^−1^). For lower inoculation doses virus amplification by infected mallards was required. Overall, the kinetics of viral RNA in pool water followed the patterns described for the mallard swab samples.

**Table 2.** Detection of viral RNA by generic RT-qPCR in water samples of infection experiments 1 (a) and 2 (b) and (c) detection of viral infectivity in selected RT-qPCR-positive water samples obtained from experiments 1 and 2.

(a)											
Days *p*.i.											
	0	1	2	3	4	5	6	7	8	9	11
LP bell drinker	neg	neg	22.10	20.15	18.55	18.16	19.93	23.25	25.11	25.17	26.25
LP pool	neg	neg	34.44	neg	neg	27.59	20	24	19.48	23.72	25.36
HP bell drinker	neg	neg	29.18	34.25	neg#	29.37	26.65	27.11	neg	neg	n.e.
HP pool	neg	25.54	29.08	25.04	25.28	23.43	24.77	26.17	30.85	32.36	36.01

(b)													
Days *p*.i.													
	0	1	2	3	4	5	6	7	8	9	11	12	13
HP 100	neg	neg	neg	36.06	neg	neg	37.75	28.17	27.41	34.44	neg	neg	neg
HP 1,000	neg	neg	neg	30.87	28.09	30.90#	34.72#	34.03#	34.13#	30.599	neg	neg	neg
HP 10,000	neg	30.84	30.98	31.03	28.17	25.78	27.06	29.14	30.09	29.23	32.98	31.71	38.15

(c)			
Group/ experiment	Day *p*.i.	Cq value^a^	Virus replication^b^
LP bell drinker/1	5	18.16	++
LP pool/1	6	20	++
HP bell drinker/1	6	26.65	- -
HP pool/1	6	24.77	+-
HP 100/2	7	28.17	- -
HP 10,000/2	5	25.78	++

^a^ Cq value of the original sample as measured by generic RT-qPCR targeting the NP gene.

^b^ + indicates virus-positive embryonated chicken egg (two eggs per sample used).

Neg – Cq value ≥40; n.e. – not examined; # RNA extraction by Macherey-Nagel NucleoMag® VET-Kit. All other samples were extracted by the Zymo Environ Water RNA Kit.

## Discussion

Mallards (*Anas platyrhynchos*) are the most abundant anseriform migratory wild bird species in many regions of Europe. They are occupying an important role as reservoir species in the maintenance and spread of LPAI viruses [2,51]. They have been implicated also at least in regional, but possibly also long distance, spread of HPAI viruses following spill-over events from infected poultry [52,53]. The importance of abiotic factors in the spread and perseverence of AIV in the environment has been pointed out repeatedly [27,54–56]. Here we combined animated and abiotic factors in controlled infection experiments aiming to study the role of water bodies in the epidemiology of AIV.

**Figure 5.**
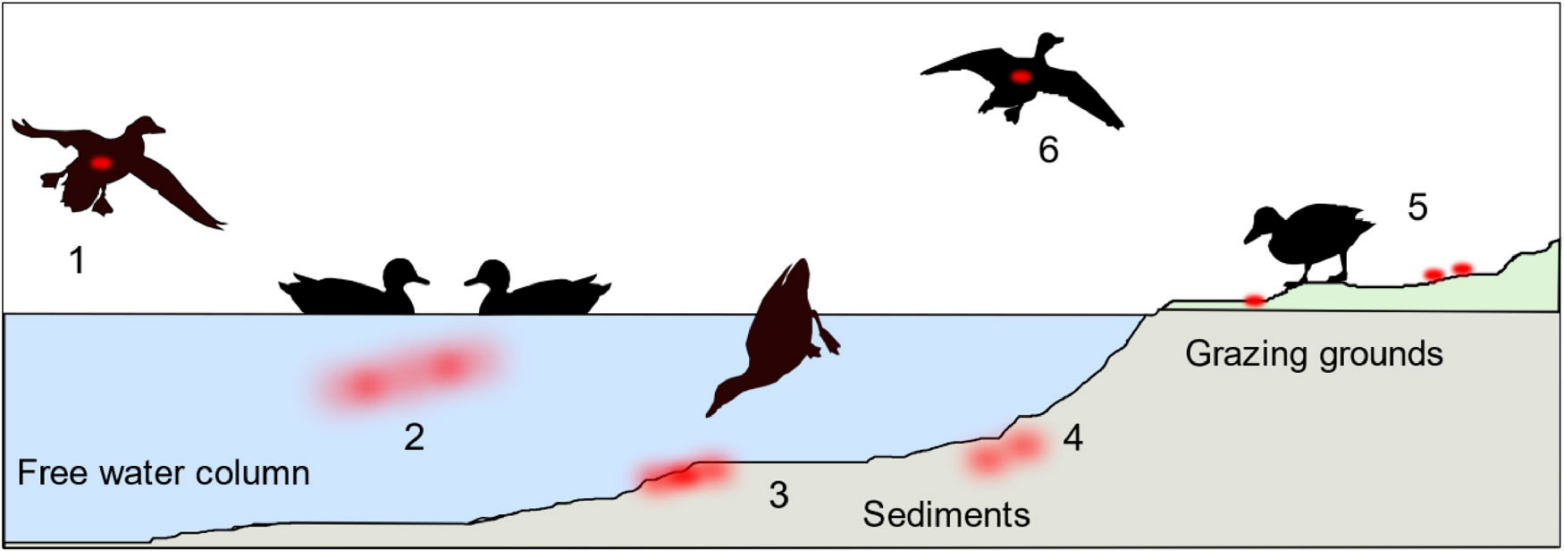
Schematic view of entry (1), dispersal (2–5) and exit (6) of avian influenza viruses within a waterbird habitat by infected aquatic wild birds. Red dots/clouds symbolize infectious virus.

Figure 5 (nos. 1–4) provides a mechanistic synthesis of some of the possible drivers of AIV infection in this respect: AIV-infected mallards land on surface waters and excrete virus into the water which disperses and might float in the water column or eventually sediment. This situation is mimicked by our experiment 1, in which two inoculated seeder ducks were used as a source of infection of eight contact mallards. Using detecting excretion of AIV RNA and seroconversion, we showed that LP as well as HP virus transmitted successfully and initiated productive infection in naïve contact mallards both in the presence and absence of a small pool resembling shallow surface water bodies. In this respect, the seeder duck concept proved successful; virus transmissions in such an experimental setting more closely resemble natural infections of ducks as compared to the standard oculo-oronasal inoculation. As the infection started to transmit and progressed in the contact ducks, slight differences in the dynamics of virus excretion became detectable between the pool and the bell drinker groups. The LP pool group in particular was slow to start the infection but eradicated the virus faster as shown by receding virus excretion. Causes for delayed onset of infection in the wet housing surrounding were not due to slowed progression of infection of the seeder ducks of the different groups. Instead, the larger volume of water available in the pool group might have had a diluting effect on virus available for infection of sentinel ducks. In contrast, the very limited volume of water accessible at a time in the bell drinker might have provided higher-titred virus inocula. This is at least suggested by the high virus loads detected over a long period in the LP bell drinker group. Yet, a long period of moderate to high viral RNA loads was also recorded for the HP pool group of experiment 1 (Table 2). In addition, using selected water samples from both bell drinker and pool groups we demonstrated the presence of AIV infectivity.

As shown in Figure 5, no. 5, other AIV-negative, naïve mallards may access surface water that had been virus-contaminated before. Experiment 2 establishes this situation in a stable unit where pool water was artificially loaded with infectious HPAIV. Surprisingly low titres of infective HP virus of 10^2^ TCID_50_ per litre water were sufficient to set off an infection in all of the exposed mallards within four to five days of observation. Infection progressed rapidly through the groups of four mallards once the first bird had caught the infection and served itself as a virus seeder. Progression of infection in the group may well have been achieved through other contacts than surface water.

Transposure of AI viruses from contaminated surface waters over wider geographic regions is most easily conceivable via infected migrating birds (Figure 5, no. 6). This requires that mobility is retained in the infected birds. In our experiments 1 and 2, very few clinical signs could be verified and only three out of 32 mallards succumbed to a neurological manifestation of the HPAIV infection. There are field investigation data suggesting that aquatic birds, in particular dabbling ducks, may also carry AIV infectivity in their plumage while accessing contaminated water bodies [57]. “Charging” the plumage in one water body and unloading it into another one would help spreading virus effectively. We showed that considerable amounts of viral RNA are present on surface plumage swabs of infected mallards and that at least some of them harbour viral infectivity. Our findings seem to indicate that the presence of virus at the breast plumage is likely due to plumage care/preening as hardly any differences in the viral RNA loads in the plumage of the pool versus bell drinker groups was evident. In our experimental setting 3, however, we were unsuccessful to demonstrate virus transmission via putatively “charged” plumage of uninfected mallards. Failure may be due to a limited amount of virus to effectively load the plumage or due to insufficient contact times to the sentinel due which hindered proper unloading of substantial virus amounts.

In conclusion, considering the environment, and water bodies in particular, as a potential source of infection of aquatic wild birds is important. Quantifying the risks that emanate from such sources, however, will remain challenging since many factors influence amount, stability, and availability of viruses to susceptible hosts. We showed that astonishingly low titres of HPAI virus that even escaped RT-qPCR detection, when dispersed in surface water, started an infection in mallards. Virus exposure and infection in such settings may be rare stochastic events but once a single bird enters productive infection, the likelihood of spread increases exponentially.

## Supplementary Material

Supplemental Material

## Data Availability

All data pertinent to this study are presented in tables and figures in the main text or in the supplementary materials.

## Software

Figure 1 was created with BioRender.com and licensed by the company under agreement number VP23LU0PN1. Other figures were created with GraphPad Prism software version 7 (GraphPad Software, San Diego, CA, USA) or with Inkscape version 1.01.
