# Supplementary material for: Exploring surface water as a transmission medium of avian influenza viruses – systematic infection studies in mallards
Source: Emerg Microbes Infect. 2022 May 3;11(1):1250–61. doi: 10.1080/22221751.2022.2065937 (PMC9090351; doi:10.1080/22221751.2022.2065937)

**Supplemental Material**

**Supplemental figure 1.** Clinical scores of “seeder” ducks (black squares) inoculated with LPAIV H4N6 (a, b) or HPAIV H5N8 (c, d) and of exposed contact mallards (open circles) housed in the presence of a small swimming pool (a, c) or of a bell drinker only (b, d).


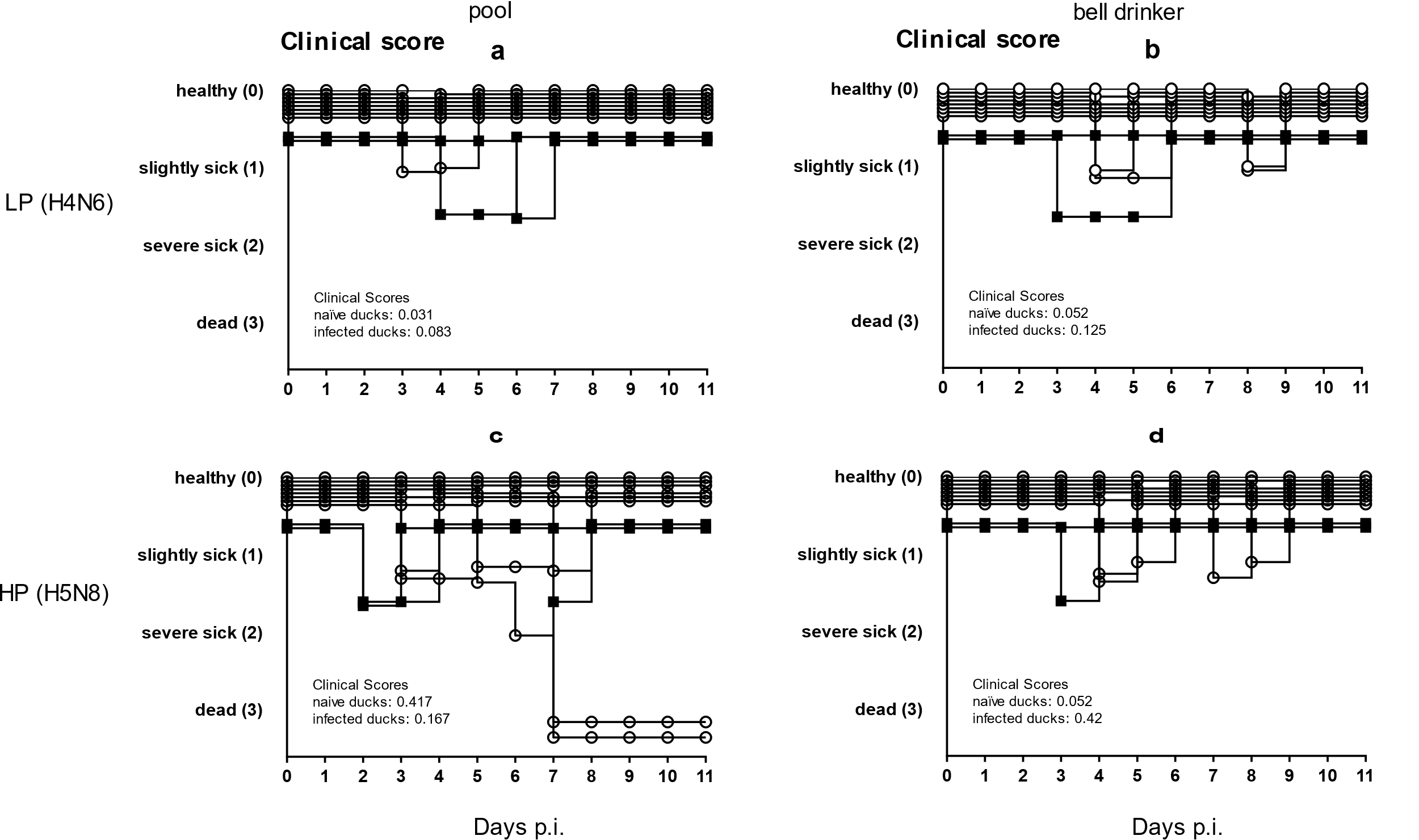


**Supplemental figure 2.** Clinical scores of mallard ducks exposed to pool water artificially loaded with HPAIV H5N8 at (a) 10^2^, (b) 10^3^ or (c) 10^4^ TCID_50_ L^-1^ or to a “carrier duck” with virus-contaminated plumage (d).


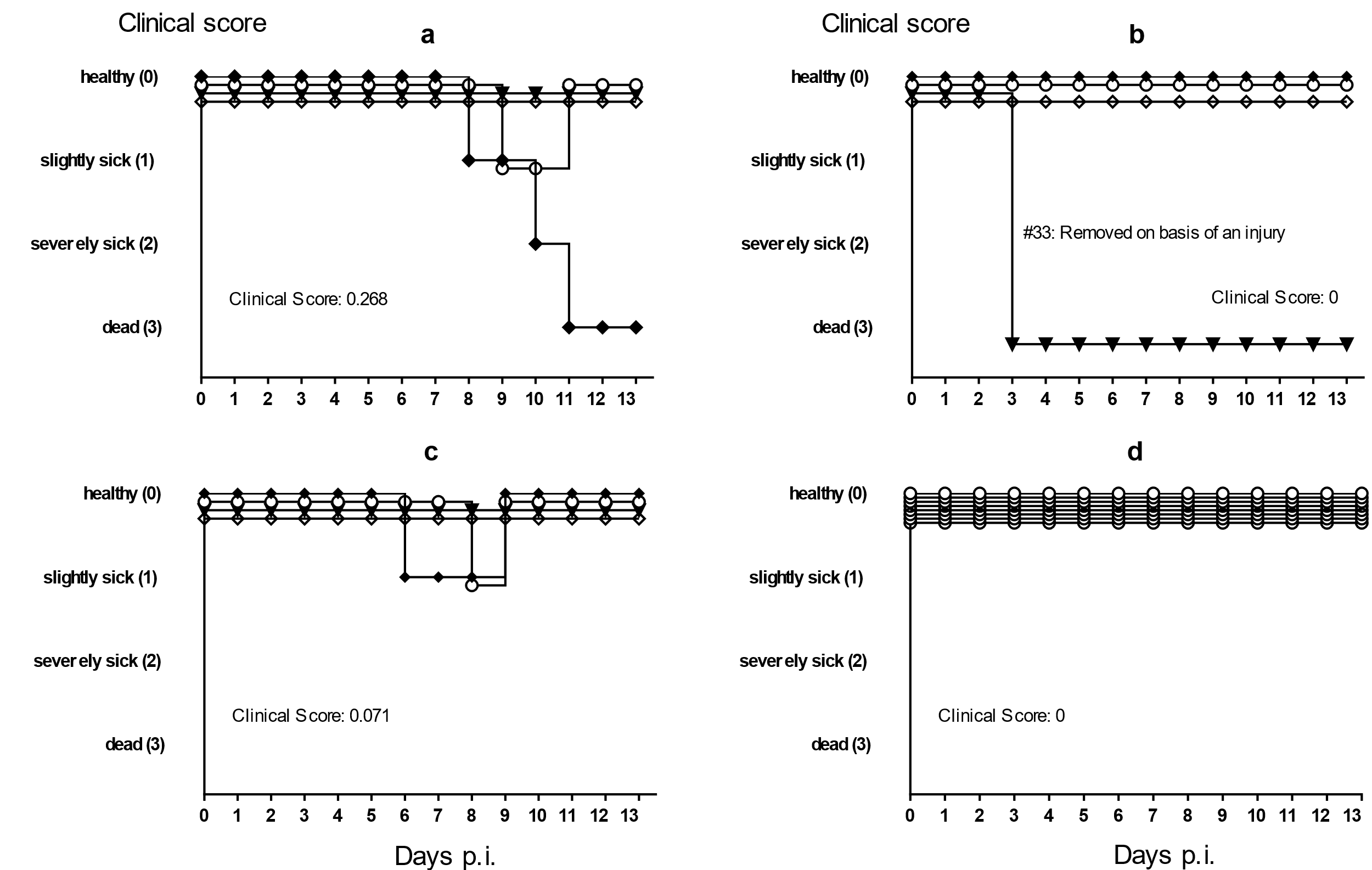

Supplement: Supplemental Material [file TEMI_A_2065937_SM3119.docx]
